# Neurodevelopmental outcomes in adolescents born very and extremely preterm: a prolonged follow-up from a single-center cohort

**DOI:** 10.3389/fped.2026.1861045

**Published:** 2026-06-22

**Authors:** Emanuela Claudia Turco, Laura Caiazza, Martina Gnazzo, Valentina Baldini, Giulia Pisanò, Cristel Brugnano, Lorenzo Petrolini, Tommaso Vitali, Benedetta Piccolo, Beatrice Campana, Susanna Esposito, Matteo Tonna, Maria Carmela Pera

**Affiliations:** 1 Child Neuropsychiatry Unit, Department of Medicine and Surgery, University of Parma, Parma, Italy; 2 Department of Biomedical, Metabolic and Neural Sciences, University of Modena and Reggio Emilia, Modena, Italy; 3 Department of Biomedical and Neuromotor Sciences, University of Bologna, Bologna, Italy; 4 Department of Medicine and Surgery, Psychiatric Unit, University of Parma, Parma, Italy; 5 Pediatric Clinic, University Hospital, Department of Medicine and Surgery, University of Parma, Parma, Italy; 6 Department of Mental Health, Local Health Service, Parma, Italy

**Keywords:** adolescents, cognitive outcome, long-term follow-up, motor development, neurodevelopment, preterm birth

## Abstract

**Introduction:**

Preterm birth remains a major global health concern, with growing evidence linking it to long-term neurodevelopmental and psychiatric sequelae. Children born very or extremely preterm are at increased risk for cognitive, motor, and emotional difficulties that may persist into adolescence.

**Materials and methods:**

We conducted a retrospective and prospective observational study involving 29 adolescents (aged 14–18 years) born very or extremely preterm between 2005 and 2011 at the University Hospital of Parma. Participants underwent in-person neurological examinations, cognitive testing (Raven's Progressive Matrices), and motor assessments (Movement ABC-2 or Brief Motor Scale), alongside retrieval of early developmental scores from the Bayley Scales (administered between 3 and 36 months).

**Results:**

Early cognitive assessments showed strong predictive associations with later intellectual functioning: MDI scores at 12 and 18 months correlated significantly with Raven QI equivalent scores in adolescence (*ρ* = 0.705 and *ρ* = 0.763, respectively, *p* < .01), while the association at 3 months was weaker and non-significant (*ρ* = 0.410, *p* = .052). In contrast, early motor scores showed inconsistent predictive associations, with only the 18-month PDI reaching significance (*ρ* = 0.583, *p* = .022). Psychiatric or neurodevelopmental diagnoses were present in 50% of extremely preterm and 33.3% of very preterm adolescents. Visuomotor coordination showed the strongest negative correlation with gestational age among motor domains (*r* = –0.57).

**Conclusions:**

These findings suggest that the 12–18 month developmental assessment window carries meaningful prognostic value for both cognitive and motor outcomes in preterm survivors, and support the implementation of structured multidisciplinary surveillance extending into adolescence.

## 1 Introduction

Prematurity, defined by the World Health Organization as birth before 37 completed weeks of gestation, affects more than 10% of newborns globally each year and is a major contributor to neonatal morbidity and mortality (World Health Organization, 2012). Preterm birth is further categorized by gestational age into extremely preterm (<28 weeks), very preterm (28–32 weeks), and moderate to late preterm (32 to <37 weeks), with corresponding classifications by birth weight. Advances in perinatal and neonatal care have improved the survival of extremely or very preterm, but as a result there has been an increased incidence of multisystem complications, including respiratory, gastrointestinal, immune, cardiovascular, and neurological dysfunctions (1). From a neurodevelopmental perspective, children born very preterm are at increased risk for cerebral palsy, cognitive impairments, attention deficits, and psychiatric disorders such as anxiety, depression, and autism spectrum disorder (2, 3). Neurological damage sustained during the neonatal period—including intraventricular haemorrhage and white matter injury—may have lasting consequences on brain structure and connectivity, thereby affecting executive functioning, processing speed, and social-emotional regulation later in life (4, 5). Current literature highlights a higher prevalence of emotional symptoms, inattention, and peer relationship problems in extremely preterm children compared to those born at term. Moreover, emerging data suggest that cognitive deficits, such as impaired executive function and reduced processing speed, may mediate the relationship between prematurity and adverse behavioral, social, and emotional outcomes in early and middle childhood (3). In severely preterm populations, the estimated prevalence of clinically significant psychiatric disorders in school-age children and adolescents ranges from 18% to 38% (3).

Despite this well-documented vulnerability, the long-term neurodevelopmental trajectory of preterm individuals into adolescence remains incompletely characterized. Most existing follow-up studies focus on outcomes at school age, leaving a relative gap in evidence regarding functioning during adolescence — a period of critical cognitive, emotional, and social development (6, 7). Furthermore, while the Bayley Scales of Infant and Toddler Development are routinely administered during neonatal follow-up to monitor early developmental progress, their value as long-term prognostic tools for cognitive and motor outcomes in adolescence has not been fully established, particularly in Southern European clinical populations.

The present study addresses this gap by describing neurodevelopmental outcomes in a cohort of adolescents born very or extremely preterm, and by examining whether early Bayley scores — obtained at 3, 12, and 18 months — predict cognitive and motor functioning in adolescence. Identifying reliable early prognostic markers has direct clinical relevance: it could support the prioritization of intensive follow-up resources toward infants most at risk of long-term difficulties, and inform the timing of early interventions.

## 2 Materials and methods

This retrospective-prospective observational study was conducted at the University Hospital of Parma (March–May 2025) in accordance with the Declaration of Helsinki and approved by the local ethics committee (Protocol 634.2024). Informed consent and assent were obtained from all participants or their guardians.

The study included 29 adolescents (aged 14–18) born very or extremely preterm (<31 weeks’ gestation) between 2005 and 2011, who were monitored over a long-term follow-up period. Participants were identified from the cohort of infants born very or extremely preterm at the University Hospital of Parma between 2005 and 2011 and previously followed within the institutional neonatal follow-up program. Inclusion in the present study required availability of early developmental data and informed consent for adolescent reassessment.

Age at follow-up assessment ranged from 13.7 to 19.5 years (mean 15.7 years). Five participants were aged 18 years or older at the time of assessment (range 18.0–19.5 years; 3 very preterm, 2 extremely preterm) and were therefore evaluated using the Brief Motor Scale (BMS) instead of the Movement ABC-2, in accordance with the age limits of each instrument.

Participants underwent a multidimensional evaluation including clinical interview, neurological examination, and cognitive and motor testing. Neurological exams were conducted by trained clinicians and interpreted qualitatively.

Cognitive functioning was assessed using Raven's Progressive Matrices. Motor skills were evaluated using the Movement ABC-2 (for those <18 years) or the Brief Motor Scale (BMS) for older adolescents. Early developmental data were retrieved from medical records, focusing on Bayley-III scores (3–36 months). Assessors were not blinded to participants’ previous assessments or clinical history.

### 2.1 Scoring procedures

All continuous test scores were converted into ordinal categories (0 = normal, 1 = borderline, 2 = pathological) for group comparisons and regression analyses, based on standardized cut-off values from the respective test manuals. Raven's Progressive Matrices and Bayley MDI/PDI scores were classified as normal (≥ 85), borderline (70–84), or pathological (< 70). Movement ABC-2 subscale and total scores were classified by percentile rank as normal (> 15th), borderline (6th–15th), or pathological (≤ 5th); for analyses, motor outcome was treated as a composite variable (normal vs. borderline/pathological). BMS scores were classified as normal (< 1.4) or pathological (≥ 1.5). CBCL/6-18 and YSR/11-18 T-scores were classified as normal (< 64), borderline (65–69), or clinical (≥ 70), following ASEBA standard scoring procedures. Detailed cut-off criteria for each instrument are reported in Supplementary Tables S1–S5.

### 2.2 Statistical analysis

Only complete-case data were analyzed; no imputation was applied. Motor outcome was treated as a composite variable (normal vs. borderline/pathological), based on standardized cut-offs from the ABC-2 or BMS. Given the exploratory nature and limited sample size, no correction for multiple comparisons was applied. Emphasis was placed on effect sizes and clinical relevance, especially for trends (*p* = .05–.10). Spearman's *ρ* was used for correlation analyses due to non-normal distribution of variables. All statistical analyses were performed using IBM SPSS Statistics (version 29.0; IBM Corp., Armonk, NY, USA) and R (version 4.3.1; R Core Team, 2023) with RStudio (version 2023.06; Posit PBC, Boston, MA, USA). Group comparisons were conducted using Fisher's exact test for categorical variables and the Mann–Whitney U test for continuous variables, given the small sample size and non-normal distributions.

## 3 Results

### 3.1 Sociodemographic data

Sociodemographic and clinical characteristics are summarized in Table 1. Males represented the majority in both subgroups, with no significant sex difference between very preterm (63%) and extremely preterm (70%) groups (*p* = 1). As expected, birth weight was significantly lower in the extremely preterm group than in the very preterm group (805 ± 181 g vs. 1247 ± 292 g, *p* = 0.006).

**Table 1:** Sociodemographic and clinical characteristics of the sample.

Variable	Very preterm (*n* = 19)	Extremely preterm (*n* = 10)	*p*-value
Male, *n* (%)	12 (63%)	7 (70%)	1.0000
Female, *n* (%)	7 (36%)	3 (30%)	1.0000
Birth weight, M	1247 ± 292	805 ± 181	**0** **.** **006**

Bold values indicate statistical significance (*p* < 0.05).

### 3.2 Neurological comorbidities

Among the 19 patients classified as very preterm, 3 presented with neurological comorbidities, accounting for 15.8% of this subgroup, while 16 patients (84.2%) had no neurological comorbidities. Neurological comorbidities included cerebral palsy (*n* = 1) and non-febrile seizures (*n* = 2).

Among the 10 patients classified as extremely preterm, none presented with neurological comorbidities. Neither febrile seizures nor headache were classified as neurological comorbidities in this study, as neither can be considered as such on the basis of symptomatology alone, in the absence of an underlying structural or neurological diagnosis.

Although neurological comorbidities appeared less frequent in the extremely preterm group (0%) compared to the very preterm group (15.8%), this difference did not reach statistical significance (*p* = 0.633).

For a detailed comparison between groups, see Table 2.

**Table 2:** Comparison of neurological and psychiatric/neurodevelopmental comorbidities between very preterm and extremely preterm patients.

Comorbidity	Very preterm	Extremely preterm	*p*-value
Neurological comorbidities, *n* (%)	3/19 (15.8%)	0/10 (0%)	0.633
Psychiatric/neurodevelopmental comorbidities, *n* (%)	6/18 (33.3%)	5/10 (50.0%)	0.444

### 3.3 Psychiatric or neurodevelopmental comorbidities

Among the 19 patients classified as very preterm, only 6 patients (33.3%) presented with psychiatric or neurodevelopmental comorbidities; data were not available for 1 very preterm patient. The reported comorbidities included learning disorders (*n* = 4), intellectual disability (*n* = 1), and anxiety (*n* = 1). One patient presented with two comorbidities. Additionally, 12 patients (66.7%) had no psychiatric or neurodevelopmental comorbidities.

Among the 10 patients classified as extremely preterm, 5 patients (50%) had at least one psychiatric or neurodevelopmental comorbidity. The reported comorbidities included intellectual disability (*n* = 3), language disorders (*n* = 2), learning disorder (*n* = 1), attention deficit disorder (*n* = 1), and anxiety disorder (*n* = 1). Two patients presented with multiple comorbidities. The remaining 5 patients (50%) had no psychiatric or neurodevelopmental comorbidities.

Although the proportion of patients with psychiatric or neurodevelopmental comorbidities was higher in the extremely preterm group compared to the very preterm group (50% vs. 33.3%), this difference did not reach statistical significance (*p* = 0.444).

For a detailed comparison between groups, see Table 2.

### 3.4 Neurological examination

Neurological examination findings were heterogeneous in both groups.

Among the 19 patients classified as very preterm, 10 patients (52.6%) had a neurological examination within normal limits. The most frequently observed abnormalities included cerebellar signs (e.g., dysmetria, tandem gait, Romberg) and gait disturbances (e.g., difficulty walking on heels or toes), each found in 5 patients (26.3%). Abnormal deep tendon or plantar reflexes were observed in 3 patients (15.8%), and reduced strength, tone, or muscle trophism in 2 patients (10.5%). Involuntary movements (e.g., clonus or tics) were also noted in 2 patients (10.5%), while dysmorphic features were documented in 1 patient (5.3%).

Among the 10 extremely preterm patients, half of the patients (50.0%) presented with a neurological examination within normal limits. The most commonly reported abnormalities were cerebellar signs—including dysmetria, impaired coordination, and instability on tandem gait or Romberg test—observed in 4 patients (40.0%). Gait disturbances (e.g., difficulty walking on heels or toes) were noted in 3 patients (30.0%), while abnormal deep tendon or plantar reflexes were found in 2 patients (20.0%). Cranial nerve involvement was described in 2 patients (20.0%), and reduced muscle strength, tone, or trophism was reported in 1 patient (10.0%). Involuntary movements, such as clonus or tics, were documented in 1 patient (10.0%). No dysmorphic features were identified in this subgroup.

**Figure 1:**
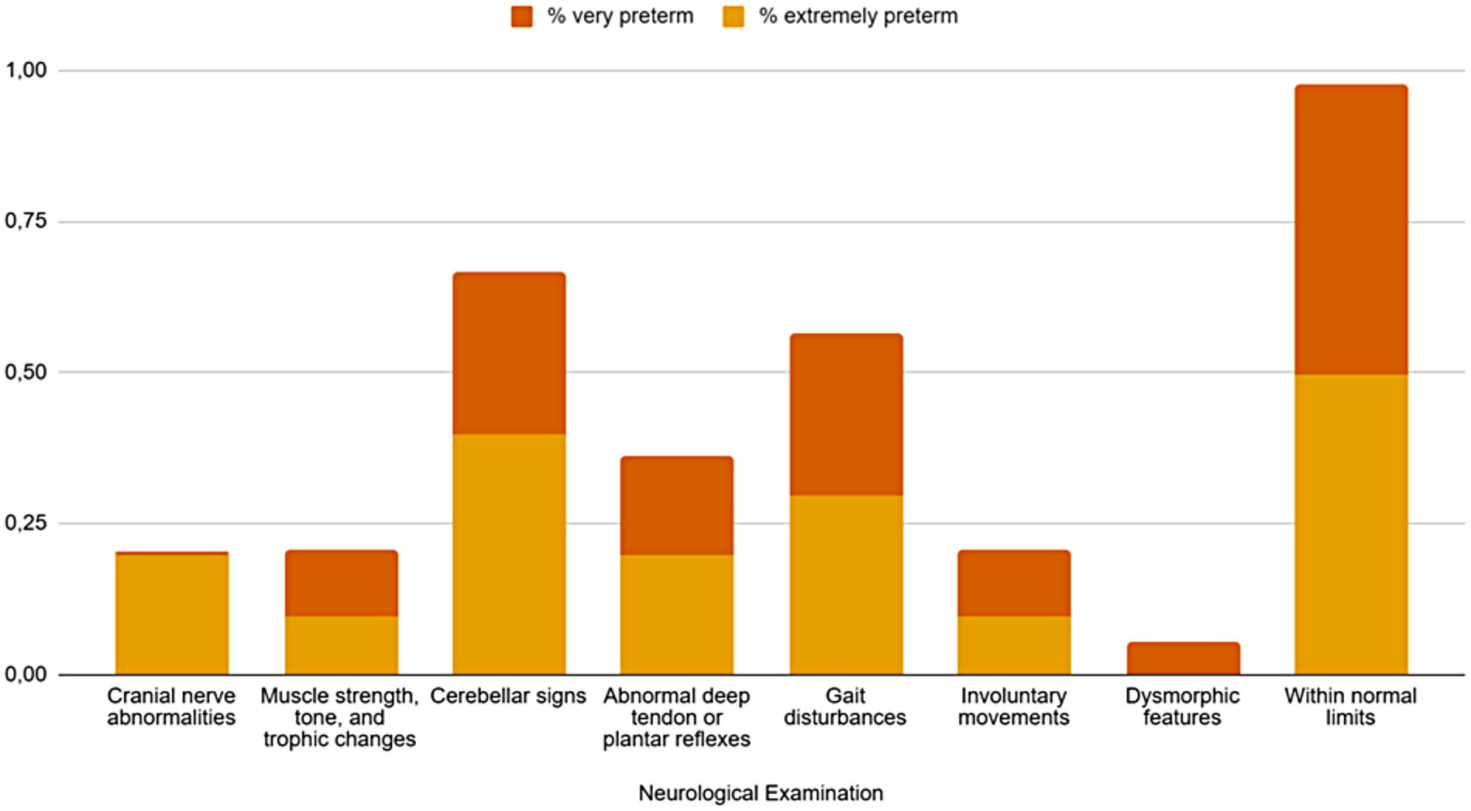
Comparison between neurological examination findings in very preterm and extremely preterm.

A detailed breakdown of neurological findings in the two groups is presented in Table 3, while a visual comparison is provided in Figure 1.

**Table 3:** Distribution of neurological examination findings among extremely and very preterm infants.

Neurological examination	Extremely preterm	Very preterm	Total (*n* = 29)
Normal examination	5 (50%)	9 (47.4%)	14 (48.3%)
Abnormal examination	5 (50%)	10 (52.6%)	15 (51.7%)

The odds of presenting at least one abnormal neurological finding were higher among very preterm infants compared to extremely preterm infants. The calculated odds ratio was 0.9 (95% CI: 0.19–4.17), suggesting a slightly higher probability of neurological abnormalities in the very preterm group. However, this difference did not reach statistical significance (*p* = 0.89), likely due to the limited sample size (Table 4).

**Table 4:** Odds ratio for abnormal neurological examination in extremely preterm vs. very preterm infants.

Comparison	Odds ratio (OR)	95% confidence interval	*p*-value
Extremely preterm vs. Very preterm	0.9	0.19–4.17	0.89

### 3.5 Cognitive functioning and motor performance

Cognitive functioning, assessed using Raven's Progressive Matrices, was available for 26 patients (17 very preterm, 9 extremely preterm). It showed a trend toward higher average scores in the extremely preterm group (M = 0.77, SD = 0.97) compared to the very preterm group (M = 0.17, SD = 0.52), approaching statistical significance (*p* = 0.05).

It should be noted that, given the ordinal 0–2 scale used (where higher scores indicate worse performance), the higher mean score observed in the extremely preterm group reflects greater cognitive difficulty, not better performance. This counterintuitive presentation of the data is addressed in the Discussion.

Motor performance, assessed via the Movement ABC-2 or Brief Motor Scale, was available for 21 patients (14 very preterm, 8 extremely preterm). Higher difficulty levels were observed in the extremely preterm group (M = 0.57 SD = 0.98) compared to the very preterm group (M = 0.5, SD = 0.85), though this difference was not statistically significant (*p* = 0.46) (see Table 5).

**Table 5:** Cognitive and motor outcomes by gestational age group.

Outcome	Very preterm	Extremely preterm	*p*-value
Cognitive outcome (Raven categorized score)	0.17 ± 0.52 (*n* = 17)	0.77 ± 0.97 (*n* = 9)	0.05
Motor outcome (ABC-2/BMS categorized score)	0.50 ± 0.85 (*n* = 14)	0.57 ± 0.98 (*n* = 8)	0.46

Values are expressed as median [interquartile range] of categorized ordinal scores (0 = normal, 1 = borderline, 2 = pathological), derived from standardized test cut-offs. Given the ordinal nature and skewed distribution of these variables, medians and IQRs are reported instead of means and standard deviations. *p*-value <0.05 was considered statistically significant.

#### 3.5.1 Correlation between early cognitive development and later cognitive outcome

To further investigate the relationship between early neurodevelopmental abilities and later cognitive outcomes, we explored the associations between specific cognitive domains assessed in infancy and those evaluated at follow-up. In particular, we examined the correlations between cognitive scores from the Bayley Scales of Infant and Toddler Development (Mental Development Index, MDI) and cognitive performance at follow-up, as measured by Raven's Progressive Matrices (QI equivalent score).

Spearman's rank correlation analyses were conducted to assess the relationship between Mental Development Index (MDI) scores at 3, 12, and 18 months and later cognitive functioning as measured by Raven's Progressive Matrices (QI equivalent score). At 3 months, the correlation approached statistical significance (*r* = 0.410, *p* = .052). At 12 and 18 months, the correlations were strong and statistically significant (*r* = 0.705, *p* = .001 and *r* = 0.763, *p* < .001, respectively) (Table 6).

**Table 6:** Spearman's rank correlation coefficients between mental development Index (MDI) scores at 3, 12, and 18 months and raven's progressive matrices (QI equivalent score).

MDI time point	Cognitive outcome	*N*	Spearman's r	*p*-value
3 m MDI	Raven (QI equivalent)	23	0.410	0.052
12 m MDI	Raven (QI equivalent)	19	0.705	**0**.**001**
18 m MDI	Raven (QI equivalent)	20	0.763	**0**.**000**

All correlations were positive. Correlations at 12 and 18 months were statistically significant, while the association at 3 months approached significance (*p* = 0.052).

Bold values indicate statistical significance (*p* < 0.05).

Group comparisons were conducted to explore whether early cognitive performance, as measured by MDI scores at 3, 12, and 18 months, differed by sex or gestational age category (extremely vs. very preterm). No statistically significant differences were found in MDI scores by sex across any of the timepoints, although a trend toward higher scores at 3 months was observed in males compared to females (*p* = .054). When comparing gestational age groups, a statistically significant difference was observed at 18 months (*p* = .025), with higher MDI scores in very preterm children (Table 7).

**Table 7:** Group comparison for MDI by sex and gestational age.

Variable	Group comparison	N group 1	N group 2	*p*-value
3 m MDI	Extremely vs. Very preterm	9	17	0.133
3 m MDI	Male vs. Female	16	10	0.054
12 m MDI	Extremely vs. Very preterm	8	14	0.170
12 m MDI	Male vs. Female	14	8	0.165
18 m MDI	Extremely vs. Very preterm	9	13	**0**.**025**
18 m MDI	Male vs. Female	15	7	0.501

Group comparisons of mental Development Index (MDI) scores at 3, 12, and 18 months by sex and gestational age category. Group 1 and Group 2 indicate the number of subjects in each category of the comparison. For gestational age, Group 1 = extremely preterm and Group 2 = very preterm. Sample size varied depending on the availability of each variable.

Bold values indicate statistical significance (*p* < 0.05).

A multiple linear regression analysis was performed to evaluate the predictive value of early MDI scores (3, 12, and 18 months), sex, and gestational age on later cognitive outcome, operationalized as the Raven QI equivalent score. The overall model was not statistically significant; however, MDI at 18 months approached significance as an independent predictor (*β* = 0.584, *p* = .103), suggesting a potential role of late infancy cognitive performance in forecasting later intelligence. None of the other variables, including sex and gestational age, showed significant predictive power in the model (Table 8).

**Table 8:** Multiple linear regression predicting raven score.

Predictor	Coefficient (β)	*p*-value
constant	0.136	0.674
3 m MDI	−0.241	0.600
12 m MDI	0.589	0.302
18 m MDI	0.584	0.103
Sex	−0.304	0.366
Gestational age	0.209	0.541

Multiple linear regression analysis predicting Raven's Progressive Matrices (QI equivalent) score from Mental Development Index (MDI) scores at 3, 12, and 18 months, sex, and gestational age. Coefficients (β) and corresponding *p*-values are reported for each predictor. The analysis was conducted in *N* = 17 subjects with complete data for all included variables.

Note: Table 7 and Table 8 were based on *N* = 17 participants with complete data for MDI at 3, 12, and 18 months, Raven score, sex, and gestational age. Sample sizes in Table 6 vary by timepoint and are reported in the corresponding column.

#### 3.5.2 Correlation between early psychomotor development and later motor performance

To further investigate the relationship between early neurodevelopmental abilities and later outcomes, we explored the associations between specific motor domains assessed in infancy and those evaluated at follow-up. In particular, we examined the correlations between motor scores from the Bayley scales and subsequent motor outcomes measured using the Movement Assessment Battery for Children-2 (Movement ABC-2) and the Brief Motor Scale (BMS), depending on data availability.

Spearman's correlation analyses were conducted to examine the association between early cognitive development, as measured by the Psychomotor Development Index (PDI) at 3, 12, and 18 months, and later motor functioning, operationalized as the Movement ABC-2 and Brief Motor Scale equivalent score. The only one positive correlation was at 18 months (*r* = 0.583, *p* = 0.022). Correlations at 3 and 12 months were not statistically significant (*r* = - 0.134, *p* = 0.584 and *r* = −0.134, *p* = 0.635, respectively) (Table 9).

**Table 9:** Correlation between PDI and motor outcome.

PDI time point	Motor outcome	*N*	Spearman's *ρ*	*p*-value
3 months	ABC or BMS composite	19	−0.134	0.584
12 months	ABC or BMS composite	15	−0.134	0.635
18 months	ABC or BMS composite	15	0.583	**0**.**022**

Spearman's rank correlations between Psychomotor Development Index (PDI) scores at 3, 12, and 18 months and motor outcome assessed via Movement ABC-2 or Brief Motor Scale (composite score). Pairwise deletion was used to include all available data; sample size varied by timepoint. A statistically significant correlation was found at 18 months (*ρ* = 0.583, *p* = 0.022), whereas earlier timepoints showed no significant associations.

Bold values indicate statistical significance (*p* < 0.05).

Group comparisons based on gestational age (extremely preterm vs. very preterm) and sex revealed no significant differences in motor outcomes or in PDI scores at any time point. A non-significant trend toward higher PDI scores at 3 months was noted among male participants (*p* = .099), but this difference did not persist at later time points (Table 10).

**Table 10:** Group comparisons by gestational age and sex.

Variable	Group comparison	N Group 1	N Group 2	*p*-value
Motor outcome	Extremely vs. Very preterm	6	12	0.613
PDI 3m	Extremely vs. Very preterm	9	17	0.627
PDI 12m	Extremely vs. Very preterm	9	14	0.679
PDI 18m	Extremely vs. Very preterm	8	13	0.381
PDI 3m	Male vs. Female	16	10	0.099
PDI 12m	Male vs. Female	15	8	0.956
PDI 18m	Male vs. Female	14	7	0.591
Motor outcome	Male vs. Female	11	7	0.740

Group 1 and Group 2 indicate the number of subjects in each category of the comparison. For gestational age, Group 1 = extremely preterm and Group 2 = very preterm. Sample size varied depending on the availability of each variable.

Finally, a multiple linear regression model including PDI scores at 3, 12, and 18 months, sex, and gestational age as predictors of motor outcome yielded no significant results overall. Among predictors, only PDI at 18 months approached significance (*β* = 0.763, *p* = .123), while all other variables showed no independent association with later motor outcome (Table 11).

**Table 11:** Multiple linear regression predicting motor outcome.

Predictor	Coefficient (β)	*p*-value
Constant	0.445	0.502
PDI 3m	−0.07	0.782
PDI 12m	−0.254	0.376
PDI 18m	0.763	0.123
Sex	−0.065	0.745
Gestational Age	0.142	0.445

Multiple linear regression model including PDI scores (3 m, 12 m, 18 m), sex, and gestational age as predictors of motor outcome. Analysis included *N* = 11 subjects with complete data for all predictors and the outcome.

Note: Tables 10, 11 were based on *N* = 11 participants with complete data for PDI at 3, 12, and 18 months, motor outcome (ABC or BMS), sex, and gestational age. Sample sizes in Table 9 vary by timepoint and are reported in the corresponding column.

### 3.6 Correlation between gestational age and ABC subscale scores

**Figure 2:**
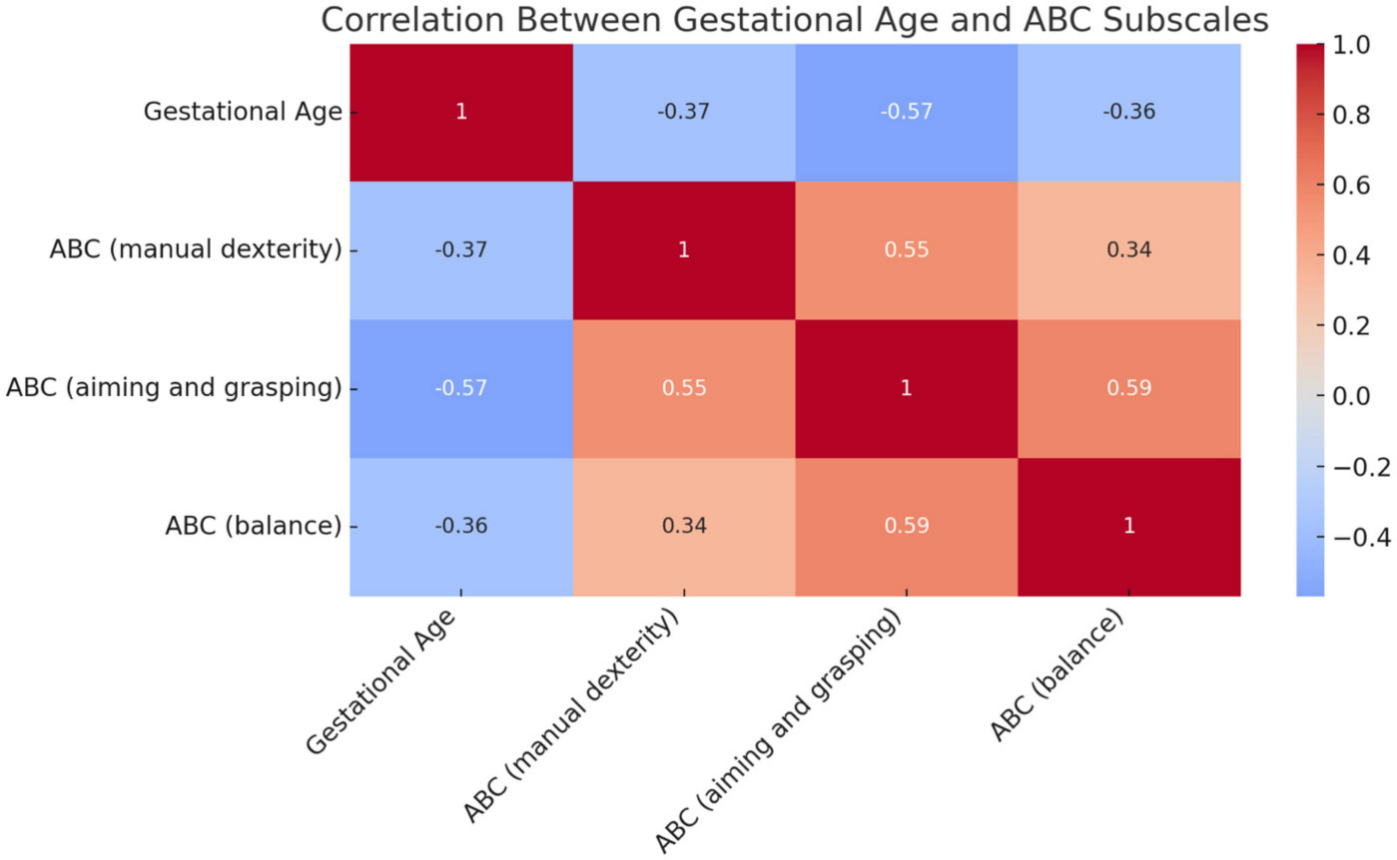
Correlation matrix between gestational Age and ABC subscales. Pearson correlation coefficients between gestational age and the three subscales of the Movement Assessment Battery for Children (ABC): manual dexterity, aiming and grasping, and balance. A negative correlation was found between gestational age and all motor subdomains, indicating that lower gestational age (i.e., greater prematurity) was associated with lower motor scores, particularly in the domains of aiming and grasping (*r* = −0.57) and manual dexterity (*r* = −0.37).

A correlation analysis was performed to examine the relationship between gestational age (coded as 0 = extremely preterm, 1 = very preterm) and motor performance based on ABC subscale scores. The heatmap (Figure 2) illustrates Pearson correlation coefficients.

- Manual dexterity: *r* = –0.37
- Aiming and grasping: *r* = –0.57
- Balance: *r* = –0.36

All coefficients are negative, suggesting that lower gestational age (i.e., more extreme prematurity) is associated with poorer performance in motor subdomains. The strongest negative correlation was found in the *aiming and grasping* subscale, indicating that visuomotor coordination may be particularly affected in extremely preterm children. Similar trends were observed for manual dexterity and balance.

## 4 Discussion

The present study adds to the growing body of evidence on long-term neurodevelopmental outcomes in preterm survivors by demonstrating that cognitive assessments conducted at 12 and 18 months of age carry meaningful prognostic value for intellectual functioning in adolescence. For clinicians involved in neonatal follow-up, this finding suggests that the second year of life represents a critical and informative window: a child who shows cognitive difficulties on the Bayley Scales at 12–18 months should be regarded as at elevated risk for persistent intellectual difficulties and prioritized for structured long-term surveillance.

As expected, extremely preterm infants showed significantly lower birth weights than their very preterm counterparts, consistent with the known relationship between gestational age and perinatal morbidity (6, 7). Approximately half of participants in both groups showed abnormal findings on neurological examination, with cerebellar signs and gait disturbances being the most frequently observed abnormalities — a pattern consistent with the lasting neurological impact of white matter injury and intraventricular hemorrhage in preterm survivors (8, 9, 19).

Notably, the prevalence of neurological comorbidities was lower in the extremely preterm group (0%) than in the very preterm group (15.8%), a finding that runs counter to the established gestational age gradient reported in the literature, where greater prematurity is typically associated with higher rates of neurological sequelae. This result warrants careful discussion and should not be interpreted at face value.

Several explanations may account for this apparent paradox. First, selection bias is likely the most important factor: children born extremely preterm who survived to adolescence and were available for follow-up represent a highly selected subgroup, from which the most severely affected individuals — those with the greatest neurological burden — may have been excluded due to early death, loss to follow-up, or inability to participate in assessment. Second, the extremely small sample size of the EP subgroup (*n* = 10) renders the 0% figure statistically fragile; a single additional case with a neurological diagnosis would substantially alter the proportion. Third, the reclassification of febrile seizures and headache as non-neurological comorbidities — which is methodologically justified, given that neither can be attributed to a structural neurological cause on the basis of symptomatology alone — further reduced the apparent comorbidity rate in this group. Finally, improvements in neonatal care over the study period may have partially attenuated the neurological impact of extreme prematurity in more recently born cohorts. Taken together, these considerations suggest that the lower observed prevalence of neurological comorbidities in the EP group most likely reflects methodological and sampling limitations rather than a true biological phenomenon.

The higher prevalence of psychiatric and neurodevelopmental diagnoses in the extremely preterm group (50% vs. 33.3%), though non-significant (*p* = 0.444), is consistent with prior literature documenting elevated rates of intellectual disability, language disorders, and attentional difficulties in this population (10). From a clinical perspective, the profile observed in our extremely preterm participants — combining intellectual disability, language disorders, and anxiety — mirrors the complex, multi-domain neuropsychiatric presentation that neurologists and child neuropsychiatrists are increasingly encountering in adolescent preterm survivors, and underscores the need for comprehensive psychiatric evaluation as part of long-term follow-up protocols.

A counterintuitive finding was the trend toward higher Raven scores in the extremely preterm group compared to the very preterm group (*p* = 0.05). The Raven's Progressive Matrices assess fluid intelligence and visuospatial reasoning — domains that may be relatively preserved even in the context of broader neurodevelopmental difficulties. However, this result is most plausibly attributable to the same survivor bias discussed above, as those with the most severe cognitive impairments were likely underrepresented in the follow-up sample. The small and unequal subgroup sizes further limit the reliability of this comparison, and the finding should not be interpreted as evidence of a genuine cognitive advantage associated with greater prematurity.

Regarding the predictive value of early assessments, the MDI–Raven correlation strengthened progressively from 3 months (*ρ* = 0.410, *p* = .052) to 12 months (*ρ* = 0.705, *p* = .001) and 18 months (*ρ* = 0.763, *p* < .001), suggesting that cognitive assessments gain prognostic validity as the infant matures (11). This pattern has a direct clinical implication: a single early assessment at 3 months is insufficient to characterize a child's cognitive trajectory, whereas assessments at 12 and 18 months provide substantially more reliable prognostic information. In regression models, MDI at 18 months showed the strongest independent association with later cognitive scores (*β* = 0.584, *p* = .103), further supporting the prognostic primacy of this timepoint (12, 20). It should be acknowledged that correlation coefficients derived from small samples are inherently unstable and may overestimate the true population effect; replication in larger cohorts is therefore necessary before these estimates can be considered reliable. These analyses capture statistical associations rather than formal predictive validity; estimating sensitivity and specificity would require larger samples. Nevertheless, the consistency and strength of the observed correlations across two independent timepoints supports their potential clinical relevance.

Regarding motor development, only the 18-month PDI correlated significantly with follow-up motor scores (*ρ* = 0.583, *p* = .022), reinforcing the clinical importance of the 18-month assessment window and aligning with prior evidence that very early motor assessments have limited long-term validity (13). Among the Movement ABC-2 subscales, visuomotor coordination showed the strongest negative correlation with gestational age (*r* = –0.57), identifying this domain as particularly vulnerable in extremely preterm children and deserving of targeted monitoring and early rehabilitation referral (14, 15).

Taken together, these findings support a model of preterm follow-up in which the 12–18 month developmental assessment is treated not merely as a snapshot of current functioning, but as a prognostically meaningful data point guiding decisions about the intensity and duration of surveillance. Given the complex and multi-domain neuropsychiatric profile observed in our cohort — spanning cognitive, motor, linguistic, and emotional difficulties — follow-up programs should be multidisciplinary by design, integrating neurology, neuropsychology, child psychiatry, and rehabilitation. This is particularly relevant for extremely preterm survivors, who appear to carry a disproportionate burden of psychiatric and neurodevelopmental morbidity into adolescence despite appearing neurologically intact on clinical examination.

**Figure 3:**
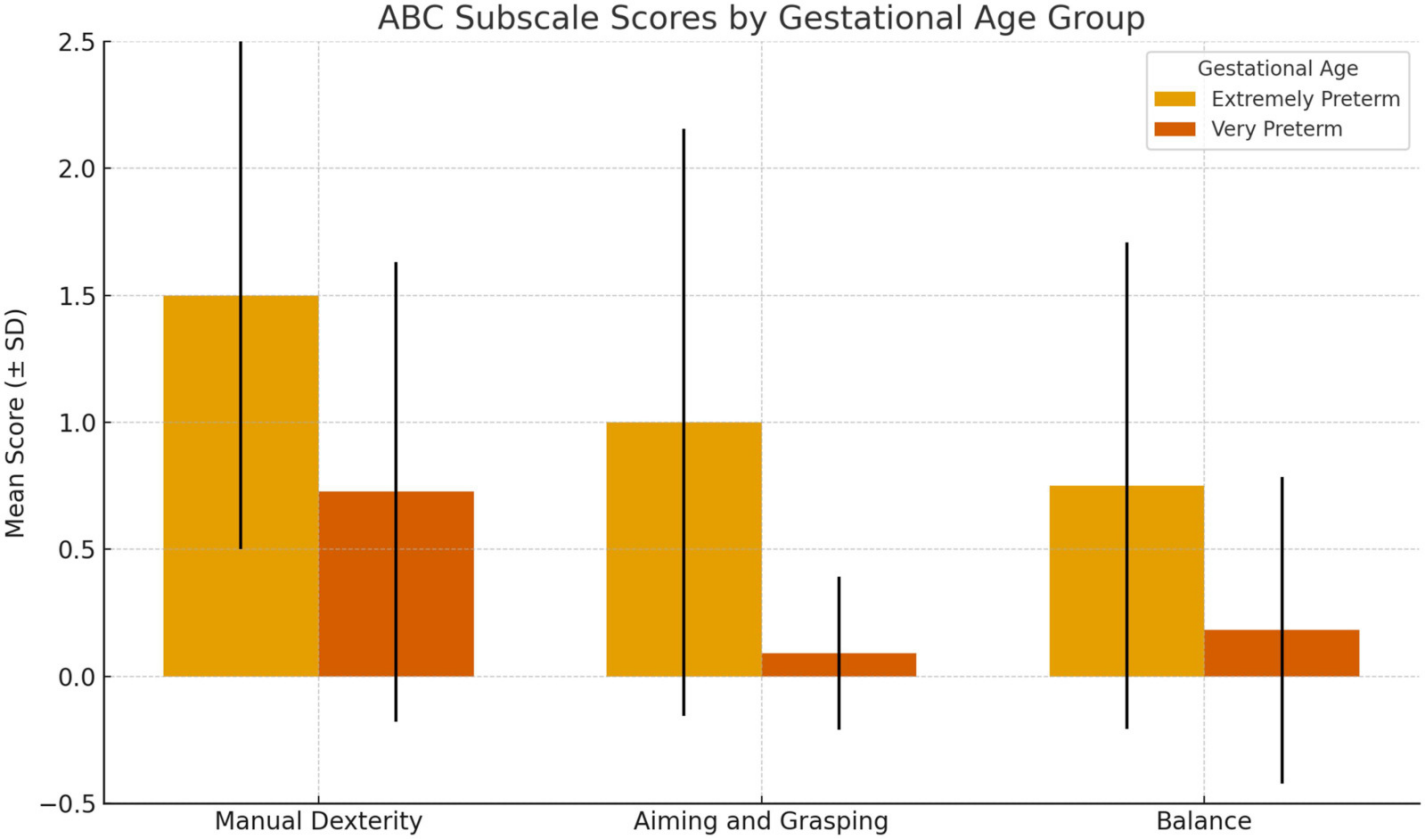
Mean ABC subscale scores by gestational Age group. Bar plot showing mean motor subscale scores (± SD) on the Movement Assessment Battery for Children (ABC) by gestational age group: extremely preterm (orange) vs. very preterm (dark orange). Children born extremely preterm had lower scores across all subdomains—manual dexterity, aiming and grasping, and balance—suggesting greater motor difficulties compared to those born very preterm.

The apparently higher cognitive scores in the extremely preterm group observed in Table 5 and Figure 3 deserve clarification. On the ordinal scale used (0 = normal, 1 = borderline, 2 = pathological), higher values reflect worse performance. Accordingly, the trend toward higher scores in the EP group is consistent with greater cognitive difficulty, not superior functioning. However, this result should be interpreted with extreme caution given the very small sample sizes, the skewed distribution of scores, and the ordinal — rather than continuous — nature of the outcome variable. The use of medians and interquartile ranges, rather than means and standard deviations, is more appropriate for this type of data and has been adopted in the revised Table 5.

This study has several strengths, including its longitudinal design spanning infancy to adolescence, the integration of cognitive, motor, and neurological domains within a single cohort, and the use of standardized validated instruments at follow-up. The retrospective retrieval of Bayley scores from clinical records allowed for a rare long-term perspective on developmental trajectories that is seldom achievable in routine clinical settings.

Several limitations must be acknowledged. The sample is small (*n* = 29) and derives from a single centre (University Hospital of Parma, Italy), which substantially limits both statistical power and the generalizability of findings. Outcomes necessarily reflect the specific neonatal and follow-up care practices of this institution and may not be representative of centres with different clinical protocols or healthcare systems, including those in the United States or other high-income countries where neonatal care organisation differs substantially.

The total number of eligible preterm infants born at the University Hospital of Parma during the study period was not systematically documented in a dedicated registry, making it impossible to formally quantify selection bias. The final cohort may represent a positively selected subgroup, as inclusion depended on survival to adolescence, availability for follow-up, and participation consent. Patients who were deceased, lost to follow-up, or declined participation were not included, potentially leading to underrepresentation of individuals with more severe neurodevelopmental impairment.

Detailed sociodemographic data beyond sex and birth weight were not systematically collected, limiting comparability with other published cohorts. The absence of statistically significant sex differences in cognitive and motor outcomes in our cohort should be interpreted with caution. Prior literature has documented sex-related disparities in neurodevelopmental outcomes after preterm birth, with males generally showing greater vulnerability to cognitive and behavioural sequelae. The lack of significant findings in the present study most likely reflects insufficient statistical power, given the small and unequal sex distribution of the sample (19 males, 10 females), rather than a true absence of sex-related differences. Adequately powered prospective studies should systematically examine sex as a moderating variable.

The absence of a term-born comparison group prevents direct contextualisation of the observed outcomes relative to typical neurodevelopmental trajectories. The use of a composite motor outcome variable increased sensitivity but reduces comparability with studies employing a single standardized instrument. Finally, assessors were not blinded to participants’ clinical history, which may have introduced evaluation bias.

It is also important to acknowledge that the participants in this cohort were born between 2005 and 2011, a period during which neonatal intensive care was already advancing but preceding several subsequent refinements in clinical practice, including the wider adoption of less invasive surfactant administration, non-invasive respiratory support strategies, and optimized early nutritional protocols. It is therefore plausible that the rates of neurodevelopmental abnormality observed in our cohort overestimate those that would be found in cohorts born under current standard-of-care conditions. Continued long-term follow-up studies of more recently born cohorts are needed to determine whether improving neonatal care is translating into better neurodevelopmental outcomes in adolescence and beyond.

Future research should prioritize multicentre longitudinal follow-up in larger and more representative preterm cohorts, with inclusion of matched term-born controls and systematic collection of sociodemographic and environmental variables. Particular attention should be directed to the 12–18 month developmental window as a candidate inflection point for early prognosis. Prospective studies should adopt standardized data collection protocols that preserve continuous raw scores alongside categorical classifications, and should examine the role of sex, socioeconomic status, and neonatal neuroimaging findings in shaping long-term neurodevelopmental trajectories (6, 7, 10, 16–18).

An important contextual factor that could not be formally evaluated in the present study is access to and uptake of early intervention services. In Italy, children born preterm are typically enrolled in structured follow-up programs coordinated by child neuropsychiatry units (Neuropsichiatria Infantile) within the National Health System, which provide multidisciplinary monitoring integrating neurological, developmental, and rehabilitative assessments. Neuromotor rehabilitation and speech-language therapy are generally available free of charge from the first months of life for children with identified risk factors. However, the intensity, duration, and continuity of these services vary considerably across regions and individual centres. In the present study, we did not systematically collect data on early intervention histories — including type, frequency, and duration of therapies received between infancy and adolescence — which prevents us from quantifying the contribution of these services to the outcomes observed at 18 months and in adolescence. This represents a meaningful limitation. Future prospective studies should systematically document early intervention exposure as a covariate, in order to disentangle the relative contributions of biological risk factors, neonatal care quality, and post-discharge interventions to long-term neurodevelopmental trajectories.

Studies conducted across multiple healthcare systems — including centres in the United States, where neonatal care organisation, follow-up protocols, and early intervention systems differ from the Italian model — will be particularly valuable to establish the generalizability of the prognostic associations identified here.

## 5 Conclusion

This study suggests that the 12–18 month developmental assessment window is clinically informative for predicting both cognitive and motor outcomes in adolescence among preterm survivors, with MDI scores at these timepoints showing the strongest prognostic associations. The complex, multi-domain neuropsychiatric profile observed in this cohort underscores the inadequacy of short-term follow-up for this population, and argues for structured multidisciplinary surveillance extending into adolescence. Larger multicentre studies are needed to confirm these findings and establish evidence-based thresholds for early risk stratification.

## Data Availability

The original contributions presented in the study are included in the article/Supplementary Material, further inquiries can be directed to the corresponding authors.
