# Supplementary material for: Neurodevelopmental outcomes in adolescents born very and extremely preterm: a prolonged follow-up from a single-center cohort
Source: Front Pediatr. 2026 Jun 22;14:1861045. doi: 10.3389/fped.2026.1861045 (PMC13333775; doi:10.3389/fped.2026.1861045)
Supplement: Supplementary file 1 [file Table1.docx]

**Supplementary Material**

Supplementary Tables S1–S5 report the classification criteria applied to convert continuous test scores into ordinal categories (0 = normal, 1 = borderline, 2 = pathological) for the Raven's Progressive Matrices, Bayley Scales (MDI and PDI), Movement ABC-2, Brief Motor Scale, and CBCL/YSR, respectively. Cut-off values are based on the standardized norms provided by the respective test manuals.

**Supplementary Table S1. Classification criteria for Raven's Progressive Matrices (cognitive functioning).**

| **Score Category** | **IQ Equivalent Range** | **Coded Value** |
| --- | --- | --- |
| Normal | ≥ 85 | 0 |
| Borderline | 70–84 | 1 |
| Pathological | < 70 | 2 |

*IQ equivalent scores were derived from age-normed tables. Scores ≥ 85 were considered within normal limits.*

**Supplementary Table S2. Classification criteria for the Bayley Scales of Infant and Toddler Development – Mental Development Index (MDI) and Psychomotor Development Index (PDI).**

| **Score Category** | **Index Score Range** | **Coded Value** |
| --- | --- | --- |
| Normal | ≥ 85 | 0 |
| Borderline | 70–84 | 1 |
| Pathological | < 70 | 2 |

*The same cut-off values were applied to both MDI (cognitive) and PDI (motor) indices. Scores were assessed at 3, 6, 9, 12, 18, 24, and 36 months.*

**Supplementary Table S3. Classification criteria for the Movement Assessment Battery for Children – Second Edition (Movement ABC-2).**

| **Score Category** | **Percentile Range** | **Coded Value** |
| --- | --- | --- |
| Normal | ≥ 16th | 0 |
| Borderline | 6th–15th | 1 |
| Pathological | ≤ 5th | 2 |

*Classification applied to each subscale (manual dexterity, aiming and catching, balance) and to the total score. For analyses, motor outcome was treated as a composite binary variable (normal vs. borderline/pathological). Administered to participants aged < 18 years.*

**Supplementary Table S4. Classification criteria for the Brief Motor Scale (BMS).**

| **Score Category** | **Score Range** | **Coded Value** |
| --- | --- | --- |
| Normal | < 1.4 | 0 |
| Pathological | ≥ 1.5 | 1 |

*The BMS was administered to participants aged ≥ 18 years. The scale provides a composite motor score; no borderline category is defined in the standard scoring manual.*

**Supplementary Table S5. Classification criteria for the Child Behavior Checklist (CBCL/6-18) and Youth Self-Report (YSR/11-18).**

| **Score Category** | **T-Score Range** | **Coded Value** |
| --- | --- | --- |
| Normal | < 64 | 0 |
| Borderline | 65–69 | 1 |
| Clinical (pathological) | ≥ 70 | 2 |

*T-scores for Internalizing, Externalizing, and Total Problems scales were classified according to ASEBA standard scoring procedures. Both parent-report (CBCL) and self-report (YSR) forms were scored using the same cut-off values.*
